# FABP4 Expression in Subcutaneous Adipose Tissue Is Independently Associated with Circulating Triglycerides in Obesity

**DOI:** 10.3390/jcm12031013

**Published:** 2023-01-28

**Authors:** Óscar Osorio-Conles, Ainitze Ibarzabal, José María Balibrea, Josep Vidal, Emilio Ortega, Ana de Hollanda

**Affiliations:** 1 Centro de Investigación Biomédica en Red de Diabetes y Enfermedades Metabólicas Asociadas (CIBERDEM), Instituto de Salud Carlos III (ISCIII), Monforte de Lemos Ave. 3–5, 28029 Madrid, Spain; 2 Institut d’Investigacions Biomèdiques August Pi i Sunyer (IDIBAPS), Rosselló Street 149, 08036 Barcelona, Spain; 3 Gastrointestinal Surgery Department, Hospital Clínic de Barcelona, Villarroel Street 170, 08036 Barcelona, Spain; 4 Obesity Unit, Endocrinology and Nutrition Department, Hospital Clínic de Barcelona, Villarroel Street 170, 08036 Barcelona, Spain; 5 Centro de Investigación Biomédica en Red Fisiopatologia de la Obesidad y Nutrición (CIBEROBN), Instituto de Salud Carlos III (ISCIII), Monforte de Lemos Ave. 3–5, 28029 Madrid, Spain

**Keywords:** adipose tissue, triglycerides, obesity, transcription, FABP4, subcutaneous adipose tissue, hypertriglyceridemia

## Abstract

Hypertriglyceridemia (HTG) has been associated with an increased risk of pancreatitis and cardiovascular disease. Adipose tissue plays a major role in lipid metabolism, mobilization and distribution. We have compared the histological and transcriptomic profiles of the subcutaneous (SAT) and visceral (VAT) adipose tissues from subjects with severe obesity undergoing bariatric surgery with (Ob-HTG, *n* = 37) and without HTG (Ob-NTG, *n* = 67). Mean age and BMI were 51.87 ± 11.21 years, 45.78 ± 6.96 kg/m^2^ and 50.03 ± 10.17 years, 44.04 ± 4.69 kg/m^2^, respectively. The Ob-HTG group showed higher levels of glycosylated hemoglobin, fasting plasma glucose, high-sensitivity C-reactive protein and prevalence of hypertension. The degree of fibrosis was increased by 14% in SAT from the Ob-HTG group (*p* = 0.028), while adipocyte size distribution was comparable. Twenty genes were found differentially expressed in SAT and VAT between study groups. Among them, only SAT expression of FABP4 resulted significantly associated with circulating triglyceride levels after adjusting for other covariates and independently explained 5% of the variance in triglyceride levels in the combined model. This relationship was not found in the cohort of lean or overweight patients with normotriglyceridemia (non-Ob, *n* = 21). These results emphasize the contribution of SAT to triglyceride concentrations in obesity and indicate that FABP4 may be a potential drug target for the treatment of HTG.

## 1. Introduction

Triglycerides (TG) are the most abundant fatty molecule and the main form of lipid storage and energy in humans [1]. Elevated TG levels (i.e., hypertriglyceridemia, HTG) are very common in clinical practice with a prevalence of ~10% in the adult population [2]. HTG has been associated with an increased risk of cardiovascular disease (CVD) in epidemiological [3,4,5], genome-wide analyses [6] and mendelian randomization studies [7]. Chronic HTG is a highly prevalent lipid abnormality in subjects with visceral obesity, type 2 diabetes (T2D) and metabolic-associated fatty liver disease (MAFLD) and a known contributor to CVD mortality [8].

The cause of HTG tends to be multifactorial and implicates the combination of genetic [9] and/or other factors leading to an increased production and/or impaired clearance of triglyceride-rich lipoproteins [10,11]. HTG, together with increased free fatty acid levels, promotes atherogenic dyslipidemia with its typical components, low HDL-C concentrations, HDL-C dysfunction, and normal, or slightly increased LDL-C, with an increased number of small and dense LDL particles. HTG has been proposed as the major cause of other lipid abnormalities [12] since it may lead to delayed clearance of TG-rich lipoproteins [13,14,15,16,17,18,19,20] and the formation of small dense LDL-C [21,22].

White adipose tissue (WAT) is a highly dynamic and active metabolic organ [23] which plays a major role in lipid metabolism and controls lipid mobilization and distribution [24]. In particular, insulin resistance status, associated with high basal and reduced insulin-inhibited lipolysis rates in WAT, has been associated with elevated TG levels [25]. In addition, transcription levels of up to 135 transcripts have been associated with TG levels after adjusting for BMI in a genome-wide study in African Americans [26].

In this study, we compared adipose tissue parameters in the subcutaneous (SAT) and visceral adipose tissue (VAT) from subjects with severe obesity with normal (Ob-NTG) and hypertriglyceridemia (Ob-HTG), we identified the expression of potential genes associated with circulating TG levels and we explored this association in a cohort with a wide range of BMIs.

## 2. Materials and Methods

### 2.1. Study Participants

White adipose tissue samples were collected from 104 subjects with severe obesity successively undergoing laparoscopic bariatric surgery at the Obesity Unit of Hospital Clínic de Barcelona between April 2018 and December 2018. The study’s exclusion criteria were a history of malignancy, previous bariatric surgery, chronic inflammatory diseases, active infectious diseases, drug abuse or daily alcohol consumption >20 g. Of these, 37 had hypertriglyceridemia (Ob-HTG) and 67 had normal triglyceride levels (Ob-NTG). HTG was considered as serum TG > 150 mg/dL according to the 2018 ACC/AHA guidelines [27]. The proportion of postmenopausal women was comparable between Ob-NTG (22 out of 49, 44.9%) and Ob-HTG (14 out of 25, 56%) groups (*p* = 0.462). Anthropometric and clinical data are summarized in Table 1.

Additionally, SAT mRNA levels of FABP4 were quantified in 21 normotriglyceridemic patients without obesity (BMI < 30 kg/m^2^) undergoing abdominal surgery (non-Ob) in the same period. Non-Ob patients underwent cholecystectomy (*n* = 11), exploratory laparoscopy (*n* = 4), eventroplasty (*n* = 4), umbilical hernia repair (*n* = 1) or appendectomy (*n* = 1). Anthropometric and clinical data from control patients are summarized in Supplementary Table S1.

All anthropometric measurements were collected following standardized clinical procedures. Ethics committee approval conforming to the Declaration of Helsinki for sample collection was obtained from the Clinical Research Ethics Committee (CEIC) of Hospital Clinic de Barcelona (R120615-084, 13 October 2016). All participants provided written informed consent.

### 2.2. Clinical and Biochemical Characteristics

Hematological and biochemical parameters were determined at the Core Laboratory of the Biomedical Diagnostic Center using an Advia 2400 analyzer (Bayer Diagnostics, Tarrytown, NY, USA), as previously reported [28]. Cholesterol and TG were measured by enzymatic procedures, and HDL-C levels after precipitation with phosphotungstic acid and magnesium chloride. The presence of T2D was considered in subjects who had either fasting plasma glucose (FPG) ≥126 mg/dL, glycosylated hemoglobin (HbA1c) ≥6.5%, or were on antidiabetic treatment [29]. MAFLD was defined as the presence of hepatic steatosis on ultrasonography (US). Hepatic US was performed in all patients after 6 h fasting, by a single experienced radiologist using a clinical US scanner (Aplio i-800, Canon Medical Systems S.A., Madrid, Spain) equipped with an i8CX1 1–8-MHz curved US transducer used for conventional B-mode examination. FIB 4 score was calculated by the following formula: FIB-4 = Age (years) × AST (IU/L)/(√ALT (IU/L) × Platelet count (10^9^/L)) [30]. APRI score was calculated by the following formula: APRI = [(AST (IU/L)/ULN)/Platelet count (10^9^/L)] × 100 [31], using 40 IU/L as the value for the AST upper limit of normal (ULN). The HSI index was calculated by the following formula: HSI = 8 × [ALT (IU/L)/AST (IU/L)] + BMI (+2, if female; +2, if diabetes mellitus) [32]. The TyG index was calculated by the following formula: TyG = Ln [TG (mg/dL) × FPG (mg/dL)/2] [33]. The FLI index was calculated by the following formula: FLI = (e^0.953×loge[TG (mg/dL)] + 0.139 × BMI + 0.718 × loge[GGT (IU/L)] + 0.053 × waist circumference (cm) − 15.745)^/(1 + e^0.953 × loge[TG (mg/dL)] + 0.139 × BMI + 0.718 × loge [GGT (IU/L)] + 0.053 × waist circumference (cm) − 15.745^) × 100 [34].

### 2.3. Tissue Processing and Histology

Paired SAT and VAT samples were obtained at the time of surgery. Tissues were fixed overnight at 4 °C in 4% paraformaldehyde and processed for standard paraffin embedding. Starting at the tissue apex 3 × 3-μm-thick sections were made at a minimum of 100 μm intervals across the sample tissue. Hematoxylin and eosin staining was used to assess adipocyte morphology. Digital images were captured under an Olympus ×600 microscope (Tokyo, Japan) at 4× magnification. Adipocyte size was measured within micrographs of at least 3000 cells per sample from randomly selected fields using Adipocytes Tools, an ImageJ macro-based algorithm for ImageJ software (National Institutes of Health, Bethesda, MD, USA; http://imagej.nih.gov/ij/, accessed on 20 December 2022). Adipocyte average area and size distribution were calculated and frequency distribution analysis into bin intervals of 200 µm^2^ was performed. Sirius red staining was used for the quantification of pericellular fibrosis. Automated analysis of the captured images at 10× magnification has been carried out using the MRI Fibrosis Tool, an ImageJ macro-based algorithm, and expressed as a percentage of red staining (fibrosis)/tissue surface ratio.

### 2.4. Quantitative Real-Time PCR

Tissue samples were immediately frozen before RNA analysis. Total RNA was isolated using RNeasy Lipid Tissue Mini Kit (Qiagen, Hilden, Germany). Concentration and purity were measured using a NanoDrop 1000 spectrophotometer (Thermo Scientific, Waltham, MA, USA). Equal amounts of RNA from SAT and VAT were reverse-transcribed using the Superscript III RT kit and random hexamer primers (Invitrogen, Carlsbad, CA, USA). The reverse transcription reaction was carried out for 90 min at 50 °C and an additional 10 min at 55 °C. Expression analysis of 65 genes involved in WAT dysfunction and related to inflammation, adipogenesis, autophagy, fatty acid metabolism and oxidation, thermogenesis and glucose metabolism, cytokines and adipokines was performed in both fat depots. Real-time quantitative PCR (qPCR) was performed with a 7900 HT Fast Real-Time PCR System (Applied Biosystems, Foster City, CA, USA) using GoTaq^®^ qPCR Master Mix (Promega Biotech Ibérica, Madrid, Spain). The amplification conditions were as follows: 95 °C for 2 min, followed by 40 cycles of 95 °C for 15 s, and 60 °C for 1 min. Expression relative to the housekeeping gene RPL6 was calculated using the delta Ct (DCt) method. Gene expression is presented as the 2^(-DCt) values. The list of primers used in this study is provided in Supplementary Table S2.

### 2.5. Statistics

Continuous data with normal and non-normal distribution are expressed with arithmetic means and standard deviations (SD), or medians and interquartile ranges (IQR), respectively. Categorical variables are expressed with frequencies and proportions. The normality assumption was tested with the D’Agostino–Pearson omnibus K2 test. The Mann–Whitney U test, Welch’s *t*-test, Student’s *t*-test, or Fisher’s exact test were used when adequate to assess the magnitude of the difference between groups. Spearman rho was used to test for correlations. Comparisons on FABP4 expression levels between non-Ob, Ob-NTG and Ob-HTG subjects were performed using a Kruskal–Wallis test followed by Dunn’s multiple comparisons test. Univariate and multivariable linear general models were used to evaluate the independent effect of variables of interest on TG concentrations. Variables considered in these models were age, sex, BMI, MAFLD, pharmacological treatment with statins, the percentage of HbA1c, and creatinine and gamma-glutamyl transferase (GGT) circulating levels. Interaction terms were tested in these models.

In all cases, a two-tailed *p*-value < 0.05 was considered statistically significant. GraphPad PRISM (GraphPad Software, version 6.0, San Diego, CA, USA) and Statistical Package for Social Sciences software (SPSS, version 17.0, Chicago, IL, USA) were used to perform the analyses.

## 3. Results

### 3.1. Differences in Adipose Tissue Parameters between Ob-NTG and Ob-HTG Patients

Characteristics of the Ob-NTG and Ob-HTG groups are shown in Table 1. Overall, the mean age and BMI were 51.5 ± 9.8 years old and 44 ± 4.9 kg/m^2^, respectively. Sex distribution, age, BMI, waist:hip ratio and the proportion of patients with T2D were comparable between groups, while the glycosylated hemoglobin (HbA1c), fasting plasma glucose (FPG), high-sensitivity C-reactive protein (hs-CRP) levels and the prevalence of hypertension were higher in the Ob-HTG group. The Ob-HTG subjects had higher scores for triglyceride glucose (TyG) and fatty liver (FLI) indices despite the prevalence of MAFLD as assessed from hepatic ultrasonography remaining comparable (*p* = 0.060).

**Figure 1:**
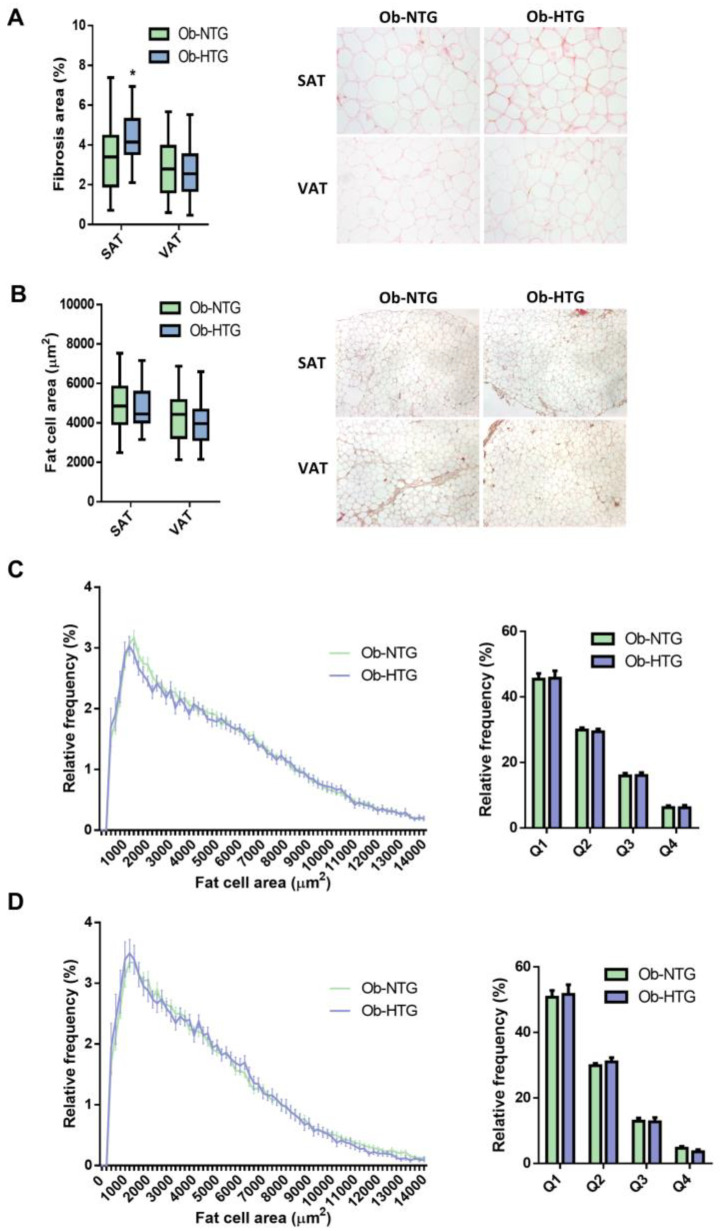
Analysis of WAT fibrosis and fat cell size distribution in Ob-NTG and Ob-HTG subjects. (**A**) Comparison of histological pericellular fibrosis and representative images. Data are presented as the mean ratio of fibrous tissue area stained with picrosirius red/total tissue surface. (**B**) Comparison of mean adipocyte area and representative images. (**C**,**D**) Frequency distribution analysis of fat cell areas divided by size into bin intervals of 200 µm^2^ and size quartiles in SAT (**C**) and VAT (**D**). Data are presented as means ± SD. Ob-NTG, subjects with severe obesity and normotriglyceridemia; Ob-HTG, subjects with severe obesity and hypertriglyceridemia; SAT, subcutaneous adipose tissue; VAT, visceral adipose tissue. * = *p* < 0.05.

The mean fibrosis area in SAT from Ob-HTG subjects was increased by 14% (*p* = 0.028) while VAT values were similar (Figure 1A). Mean SAT- and VAT-adipocyte areas did not significantly differ among groups (Figure 1B) and frequency distribution analysis in both SAT (Figure 1C) and VAT (Figure 1D) were similar even when cell sizes were separated into bin intervals of 200 µm^2^ or area quartiles.

A total of 20 genes were found differentially expressed between study groups, six of them in SAT and 12 in VAT (Table 2). In SAT from Ob-HTG individuals, the expression of fatty acid-binding protein 4 (FABP4), adipose triglyceride lipase (ATGL) and iodothyronine deiodinase 2 (DIO2) were upregulated with respect to the Ob-NTG group (all *p* values < 0.05). Conversely, Ob-HTG subjects displayed a decreased SAT expression of monoacylglycerol O-acyltransferase 1 (MOGAT1) and metalloproteinases (MMPs) 13 and 15.

**Figure 2:**
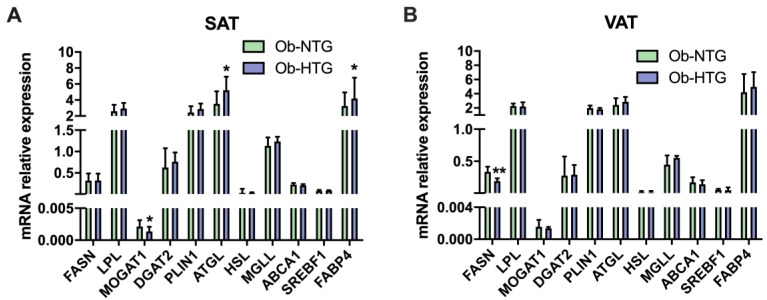
Expression analysis of genes implicated in lipid metabolism. (**A**) Gene expression levels in SAT. (**B**) Gene expression levels in VAT. Data are presented as the median (IQR). Ob-NTG, subjects with severe obesity and normotriglyceridemia; Ob-HTG, subjects with severe obesity and hypertriglyceridemia; SAT, subcutaneous adipose tissue; VAT, visceral adipose tissue. * = *p* < 0.05; ** = *p* < 0.01.

On the other hand, we found an increased expression of vascular endothelial growth factor receptor 2 (VEGFR2), adiponectin receptor 1 (ADIPOR1), insulin receptor substrate 1 (IRS1), carnitine palmitoyltransferase 1a (CPT1A) and adrenoceptors beta 1 (ADRB1) and 3 (ADRB3), in VAT from Ob-HTG group, while mRNA levels of the M1-type macrophage marker CD80, coagulation factor XIII A chain (F13A1), phosphatase and tensin homolog (PTEN), angiopoietin 1 (ANGPT1), platelet derived growth factor receptor beta (PDGFRB) and fatty acid synthase (FASN) were lower. The expression levels in SAT and VAT of other genes related to lipid metabolism are shown in Figure 2.

### 3.2. Associations between Gene Expression and Triglyceride Levels in Patients with Severe Obesity

Among the differentially expressed genes in WAT between study groups, only SAT expression of FABP4 and VAT expression of F13A1 were associated with TG levels (Table 3). After adjustment for BMI, HbA1c, MAFLD, GGT, creatinine and statin treatment, only SAT expression of FABP4 remained statistically significant (Beta = 2.83, *p* = 0.008). Additionally, SAT-FABP4 mRNA levels were associated with fatty liver index (FLI) scores (r = –0.296, *p* = 0.031) and the degree of SAT fibrosis (r = 0.286, *p* = 0.048).

### 3.3. Subcutaneous Gene Expression of FABP4 and Association with Triglyceride Levels in Subjects without Obesity and Individuals with Severe Obesity

**Figure 3:**
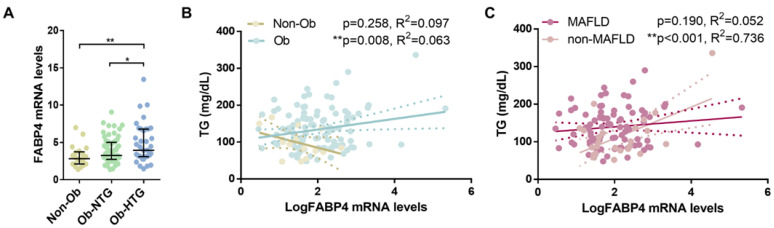
Comparison of SAT-FABP4 mRNA levels between groups and regression analysis with circulating TG levels. (**A**) FABP4 expression in SAT from normotriglyceridemic non-obese (Non-Ob), normotriglyceridemic obese (Ob-NTG) and hypertriglyceridemic obese (Ob-HTG) individuals. Data are presented as median with interquartile range. Kruskal–Wallis H(3) = 10.32, *p* = 0.005. (**B**) Simple regression analysis between SAT-FABP4 expression and TG levels in subjects with (Ob) and without obesity (non-Ob). Scatterplot presents crude, non-adjusted, relationships. (**C**) Regression analysis between SAT-FABP4 expression and TG levels in obese subjects with or without MAFLD. Simple linear regression (solid line) and 95% confidence interval (dashed line) are shown. *p*-values are adjusted for BMI, HbA1c, creatinine, GGT and statin treatment. * = *p* < 0.05; ** = *p* < 0.01.

FABP4 mRNA levels were also quantified in SAT samples from 21 normotriglyceridemic subjects without obesity (non-Ob). Thirteen female and eight male patients, 60.81 ± 9.34 years old, BMI 27.76 (25.91–28.87) kg/m^2^ without metabolic comorbidities were included in this group. The expression of FABP4 increased across study groups from non-Ob to Ob-NTG and Ob-HTG (*p* = 0.005, Figure 3A). Although SAT-FABP4 mRNA levels were increased in Ob (Ob-NTG plus Ob-HTG) vs. non-Ob groups (*p* = 0.008), no interaction term was found between obesity and FABP4 expression (OBESITY × FABP4, *p* = 0.263) in the association between FABP4 and TG levels when data from the three study groups were pooled together. Nevertheless, separate group analyses showed a direct association between TG and SAT-FABP4 expression in the whole population [Ob and non-Ob, F(1, 125) = 8.59, *p* = 0.004, R^2^ = 0.066] and in subjects with obesity [F(1, 104) = 7.18, *p* = 0.008, R^2^ = 0.063], but not in the group of non-Ob subjects alone [F(1, 21) = 1.4, *p* = 0.258, R^2^ = 0.097, Figure 3B].

A hierarchical linear regression analysis to predict TG concentrations was performed among subjects with obesity (Table 4). Model 1 included SAT expression of FABP4 (R^2^ = 0.054, *p* = 0.009). The second model included only clinical variables (BMI, HbA1c, MAFLD, GGT, creatinine and statin treatment: R^2^ = 0.143, *p* < 0.001). SAT expression of FABP4 was then added to the clinical covariables in Model 3 (R^2^ = 0.194, *p* < 0.001). SAT-FABP4 expression remained significant in Model 3 (*p* = 0.008), indicating that SAT-*FABP4* independently contributed to the prediction of circulating TG levels in this population. Interestingly, we found an interaction term (MAFLD × FABP4) between the presence of MAFLD and FABP4 (*p* = 0.001) in the association with TG concentration. Further analysis of this interaction in the unadjusted and adjusted models showed that FABP4 contributed to TG concentrations in individuals without (unadjusted: R^2^ = 0.528, *p* < 0.001; adjusted: R^2^ = 0.736, *p* < 0.001) but not with (unadjusted: R^2^ = 0.002, *p* = 0.276; adjusted: R^2^ = 0.052, *p* = 0.190) MAFLD (Figure 3C).

## 4. Discussion

In the present study, we compared different WAT variables between individuals with severe obesity undergoing bariatric surgery with or without HTG. Ob-HTG, as compared with Ob-NTG subjects, displayed comparable adipocyte area distributions in both SAT and VAT depots but increased fibrosis together with a decreased capacity for extracellular remodeling at the transcription level, MMPs in SAT. Moreover, we identified a series of differently expressed genes among the Ob-HTG and Ob-NTG groups. From a clinical point of view, the Ob-HTG group had a higher presence of hypertension and increased levels of hs-CRP and HbA1c and fatty liver and insulin resistance indexes. Interestingly, we found a positive association between SAT expression of FABP4 and circulating TG levels, which was stronger in subjects without MAFLD.

Gene expression analysis showed an upregulation of CD80 -the prototypical M1 macrophage marker- and beta-adrenergic receptors ADRB1 and ADRB3 in VAT from Ob-HTG subjects. Bidirectional communication exists between the brain and WAT occurring via the sympathetic nervous system [35]. Adipose ADRBs can be stimulated by elevated sympathetic neural activity and are implicated in lipolysis, upregulation of oxidative metabolism and thermogenesis [36]. In line with this, we found an upregulation of CPT1A in VAT from the Ob-HTG group, the key enzyme in the transport of long-chain fatty acids into the mitochondria for oxidation, whose adipose expression has been positively associated with BMI [37]. On the contrary, visceral mRNA levels of FASN were downmodulated in this group. In this regard, visceral FASN mRNA levels have been previously found inversely associated with BMI and hyperglycemia [38]. More recently, the epigenetic downregulation of VAT-FASN expression has been reported in relation to increasing HbA1c levels in subjects with severe obesity [39].

The other three genes directly implicated in lipid metabolism were differentially expressed between groups in SAT. Thus, SAT expression of both ATGL and FABP4 was upregulated in the Ob-HTG group while MOGAT1 was downregulated. ATGL initiates the hydrolysis of TG to release fatty acids and reports on its expression patterns in obesity are somewhat conflicting [40]. Thus, in mixed populations of men and women, SAT-ATGL mRNA levels have been found to increase in obesity despite decreased ATGL protein content [41,42], suggesting that posttranscriptional mechanisms may modulate ATGL activity. Conversely, other studies showed increased ATGL protein content without changes in SAT mRNA levels in obese versus lean men [43], or reduced protein and mRNA levels among obese males and females in relation to insulin resistance [44]. Gender-specific differences might explain some of these discrepancies. Of note, Ob-HTG subjects despite higher TG levels displayed lower SAT mRNA levels of MOGAT1, which catalyzes the conversion of monoacylglycerols to diacylglycerols and participates thus in TG synthesis. In this sense, suppressed expression of SAT-MOGAT1 has been found in obese subjects with increased lipolytic rates and relative insulin resistance [45].

Here, we report an upregulation of SAT-FABP4 mRNA levels in Ob-HTG subjects. Despite a modest increase in the Ob-HTG group, FABP4 was the only differently expressed gene whose expression was related to circulating TGs in this population after adjusting for covariates. To explore the extent of this association we additionally quantified SAT-FABP4 expression in a group of non-obese subjects without HTG, since transcription levels of FABP4 have been previously found increased in SAT from subjects with severe obesity [46] and insulin-resistance with respect to insulin-sensitive BMI-matched obese patients [47]. In addition, circulating FABP4 was found to increase in overweight [48,49], mildly obese [50] and morbidly obese subjects [46]. In line with previous data, we found raised SAT-FABP4 expression in Ob-HTG versus Ob-NTG and non-Ob individuals.

FABP4 encodes an adipocyte intracellular lipid chaperone implicated in regulating lipid metabolism and insulin sensitivity [51] by taking part in several pathways such as lipid droplet storage, lipid oxidation, transcriptional and enzymatic regulation, cellular signaling [52], conversion of fatty acids to eicosanoids and leukotriene stabilization [53,54]. Further, it can be released from WAT during lipolysis [55]. In the last years FABP4 has emerged as a biomarker of MAFLD [56,57,58,59,60,61,62,63] and increasing evidence suggests that FABP4 may be also a causal agent and a potential molecular target for the treatment of this disease [64]. For this reason, the reduction or inhibition of FABP4 has been proposed as a promising therapeutic approach for MAFLD or end-stage liver disease such as hepatocellular carcinoma. FABP4 disruption in mouse models results in reduced basal [65,66] and stimulated lipolysis [66,67] and the administration of FABP4 inhibitors reduces plasma TGs in mice with diet-induced obesity [68]. Similarly, the knockdown or knockout of FABP4 in obese mice models showed systemic benefits on lipid metabolism; results on glucose homeostasis seem inconclusive [69,70]. Apolipoprotein E-deficient mice null for FABP4 shows reduced atherosclerotic lesions [71] and improved atherosclerosis and survival [72]. In addition, elevated circulating FABP4 levels predicted cardiovascular death in a 12-year prospective study in the general population [73].

In our hierarchical linear regression analysis, SAT-FABP4 levels resulted in an independent positive predictor of TG levels in subjects with severe obesity when adjusting for other relevant covariables and explained a 5.4% variance in TG levels. On the contrary, this was not the case in lean and overweight subjects. Of note, FABP4 plasma levels have been previously correlated to TG levels in an obese subgroup of patients with familial combined hyperlipidemia [74] and among subjects with T2D [75]. Although higher expression in SAT versus VAT has been known for a long time [76], which depot is the major contributor to its circulating levels and the exact mechanisms underlying its secretion of FABP4 remain to be explored [77]. Despite FABP4 serum levels not being determined in our study, here we report a novel association between circulating TG and its expression in SAT in obesity.

In our study, we found an interaction term between presence of MAFLD and SAT expression of FABP4 in the relationship between FABP4 expression and TG levels. The analysis of this interaction indicated that the independent association between FABP4 and TG levels was particularly stronger in subjects without MAFLD. Of note, while other authors reported positive associations between FLI scores and serum FABP4 levels [61,62], here we show a negative relationship with its expression in SAT. Since circulating FABP4 was consistently found to be raised in relation to liver disease progression [56,57,58,59,60,61,62,63] this might imply that fatty liver could contribute significantly more than SAT to FABP4 serum levels during MAFLD progression. Thus, higher VAT expression of FABP4 has been found in subjects with vs. without metabolic-associated steatohepatitis [78] and increased liver FABP4 expression has been linked to poor outcomes and/or progression of MAFLD [79]. Moreover, the production of FABP4 has also been reported in epicardial/perivascular fat, macrophages [80] and dendritic cells [81]. Again, since the potential mechanisms have not been evaluated in our study, we cannot conclude any causal relationship between FABP4, MAFLD and TG levels. Future studies are warranted to evaluate the release of FABP4 from different depots and the interaction of liver disease progression in its association with TG levels.

We acknowledge this study is not without limitations. First, the number of patients with severe obesity exceeded the number of non-obese subjects and this may have underestimated the association in the lower range of BMI. Second, circulating FABP4 levels were not quantified in this population. This precludes associations between circulating FABP4 levels and its expression in WAT and prevented establishing comparisons with previous reports on the role of FABP4 as a biomarker of disease. Third, the FABP4 protein content in SAT was not determined in this study and this prevents establishing associations between TG levels and FABP4 protein abundance. Fourth, possible differences in insulin levels between groups have not been evaluated and their potential impact on TG levels and WAT function cannot be thus ascertained. Finally, this is an observational study and mechanistic insights are not provided. This precludes drawing causal relationships from the association between FABP4, TG levels and MAFLD.

## 5. Conclusions

Taken together, these results suggest a role for WAT in modulating lipid profiles and emphasize the association between FABP4 expression and TG levels in a population with severe obesity. Moreover, our findings propose that the SAT depot is a relevant source of circulating FABP4 contributing to this association. Further research is warranted to extend the understanding of this association in relation to the degree of obesity and liver disease progression. Functional studies will help unravel the regulation of TG levels by FABP4 in obesity and outline FABP4 as a target candidate for the control of HTG.

## Figures and Tables

**Table 1:** Clinical characteristics of the study groups.

	Ob-NTG (*n* = 67)	Ob-HTG (*n* = 37)	*p*
Women	49 (73%)	25 (67%)	0.618 ^a^
Age (years)	50.03 ± 10.17	51.87 ± 11.21	0.319
Weight (kg)	117.68 ± 20.03	122.52 ± 25.24	0.209
BMI (kg/m^2^)	44.04 ± 4.69	45.78 ± 6.96	0.075
Waist:hip ratio	0.93 ± 0.08	0.96 ± 0.06	0.222
CUN-BAE index	53.01 (51.14–55.41)	54.16 (48.98–56.15)	0.398
hs-CRP (mg/dL)	0.62 (0.34–1.14)	0.87 (0.58–1.46)	0.021
FPG (mg/dL)	99 (90–113)	104.5 (96–133)	0.028
HbA1c (%)	5.6 (5.3–6.2)	6.1 (5.6–6.8)	0.002
T2D	19 (29%)	17 (46%)	0.158 ^a^
T2D medications	0 (0–1)	0 (0–2)	0.114
Insulin treatment	8 (12%)	5 (13%)	0.999 ^a^
HTN	25 (38%)	24 (66%)	0.014 ^a^
HTN medications	0 (0–1.5)	1 (0–2)	0.062
AST (IU/L)	20 (17–25)	21 (17–26)	0.653
ALT (IU/L)	22 (17–32)	24 (18–34)	0.394
GGT (IU/L)	24 (17–36)	29 (23–54)	0.001
AST:ALT ratio	0.88 (0.70–1.12)	0.80 (0.63–1.13)	0.313
MAFLD	48 (72%)	33 (90%)	0.060 ^a^
FIB4 score	0.82 (0.61–1.07)	0.82 (0.58–1.07)	0.856
APRI score	0.18 (0.14–0.24)	0.19 (0.15–0.25)	0.545
HSI index	55.25 (52.00–58.93)	56.66 (53.99–62.40)	0.055
TyG index	4.6 ± 0.21	4.99 ± 0.14	<0.0001
FLI index	97.64 (94.29–99.02)	98.5 (97.36–99.64)	0.040
Total cholesterol (mg/dL)	177.72 ± 31.83	195.29 ± 41.68	0.005
TG (mg/dL)	99 (80.25–122.50)	181 (164.5–202.5)	<0.0001
HDL (mg/dL)	48.22 ± 10.43	45.39 ± 10.27	0.123
LDL (mg/dL)	110.24 ± 27.89	115.34 ± 37.8	0.347
Hypercolesterolemia	25 (38%)	16 (43%)	0.818 ^a^
Statins treatment	18 (28%)	15 (40%)	0.335 ^a^
GFR (ml/min/1.73 m^2^)	90 (90–90)	90 (90–90)	0.270
Creatinine (mg/dL)	0.69 (0.63–0.75)	0.67 (0.62–0.80)	0.934

Data are presented as the mean  ±  SD, median (IQR) or number (%). Ob-NTG, subjects with severe obesity and normotriglyceridemia; Ob-HTG, subjects with severe obesity and hypertriglyceridemia; CUN-BAE Index, body adiposity estimator; hs-CRP, high-sensitivity C-reactive protein; FPG, fasting plasma glucose; HbA1c, glycosylated hemoglobin; T2D, type 2 diabetes; HTN, hypertension; AST, serum aspartate aminotransferase; ALT, serum alanine aminotransferase; GGT, gamma-glutamyl transferase; MAFLD, metabolic-associated fatty liver disease; FIB-4, index for liver fibrosis; APRI, AST to platelet ratio index; HSI, hepatic steatosis index; TyG, triglyceride glucose index; FLI, fatty liver index; TG, serum tri-glyceride; HDL, serum high-density lipoprotein cholesterol; LDL, serum low-density lipoprotein cholesterol; GFR, glomerular filtration rate according to the CKD-EPI equation. ^a^ Fisher’s exact test.

**Table 2:** Differently expressed genes in adipose tissue between Ob-NTG and Ob-HTG subjects.

	Ob-NTG	Ob-HTG	*p*
SAT			
FABP4	3.237 (2.732–4.942)	4.177 (3.119–6.793)	0.032
MOGAT1	0.002 (0.001–0.003)	0.001 (0.0008–0.002)	0.044
ATGL	4.192 ± 2.111	5.456 ± 2.886	0.040
DIO2	0.006 ± 0.003	0.011 ± 0.005	0.024
MMP13	0.0009 (0.0007–0.002)	0.0005 (0.00004–0.001)	0.009
MMP15	0.024 (0.015–0.030)	0.016 (0.013–0.023)	0.029
VAT			
CD80	0.015 (0.012–0.022)	0.012 (0.011–0.013)	0.045
F13A1	0.335 (0.190–0.416)	0.187 (0.123–0.220)	0.048
PTEN	1.048 ± 0.389	0.798 ± 0.196	0.001
VEGFR2	0.001 ± 0.001	0.002 ± 0.0007	0.042
ANGPT1	0.026 (0.017–0.039)	0.014 (0.012–0.022)	0.028
PDGFRB	0.002 (0.0009–0.006)	0.0007 (0.0004–0.001)	0.032
ADIPOR1	0.150 (0.100–0.191)	0.202 (0.140–0.244)	0.012
FASN	0.082 (0.076–0.099)	0.067 (0.063–0.090)	0.008
IRS1	0.109 (0.086–0.140)	0.131 (0.115–0.157)	0.017
CPT1A	0.019 (0.014–0.027)	0.027 (0.019–0.038)	0.031
ADRB3	0.156 (0.124–0.197)	0.197 (0.153–0.242)	0.006
ADRB1	0.356 (0.230–0.609)	0.512 (0.412–0.676)	0.027

Data are presented as the mean  ±  SD or median (IQR). Ob-NTG, subjects with severe obesity and normotriglyceridemia; Ob-HTG, subjects with severe obesity and hypertriglyceridemia; SAT, subcutaneous adipose tissue; VAT, visceral adipose tissue. *p* < 0.05 for all.

**Table 3:** Regression analysis between gene expression in WAT and TG levels.

	Correlation Coefficient (95%CI)	*p*	R^2^
SAT			
FABP4			
Unadjusted	2.967 (0.773, 5.162)	0.009	0.054
Adjusted 1 ^a^	2.870 (0.688, 5.053)	0.010	0.084
Adjusted 2 ^b^	3.058 (0.931, 5.184)	0.005	0.137
Adjusted 3 ^c^	2.828 (0.74, 4.92)	0.008	0.194
VAT			
F13A1			
Unadjusted	72.704 (8.8, 136.609)	0.027	0.114
Adjusted 1 ^a^	64.55 (1.813, 127.286)	0.044	0.177
Adjusted 2 ^b^	52.272 (−19.133, 123.676)	0.181	0.238
Adjusted 3 ^c^	23.816 (−30.041, 77.674)	0.293	0.033

Adjusting for: ^a^ BMI and HbA1c, ^b^ BMI, HbA1c, MAFLD, GGT and creatinine, ^c^ BMI, HbA1c, MAFLD, GGT, creatinine and statin treatment. SAT, subcutaneous adipose tissue; VAT, visceral adipose tissue.

**Table 4:** Summary of hierarchical regression analysis for variables predicting circulating TG levels in subjects with obesity.

Model	Predictor	B	SE	95% CI	*p*	Adjusted R^2^	*p*
1	Constant	128.903	4.979	(119.03, 138.77)	<0.0001	0.054	0.009
	SAT-FABP4	2.967	1.107	(0.77, 5.16)	0.009		
2	Constant	−1.561	40.706	(−82.14, 79.02)	0.969	0.143	<0.001
	BMI	2.276	0.694	(0.9, 3.65)	0.001		
	HbA1c	0.836	4.636	(−8.34, 10.01)	0.857		
	MAFLD	21.204	10.492	(0.43, 41.97)	0.045		
	Creatinine	−45.560	35.564	(−115.96, 24.84)	0.203		
	GGT	22.030	10.562	(1.12, 42.94)	0.039		
	Statins	14.440	10.280	(−5.91, 34.79)	0.163		
3	Constant	−4.613	41.243	(−86.4, 77.17)	0.911	0.194	<0.001
	SAT-FABP4	2.828	1.054	(0.74, 4.92)	0.008		
	BMI	2.108	0.72	(0.68, 3.54)	0.004		
	HbA1c	0.345	4.747	(−9.07, 9.76)	0.942		
	MAFLD	21.925	11.403	(−0.69, 44.54)	0.057		
	Creatinine	−42.67	34.886	(−111.85, 26.51)	0.224		
	GGT	15.188	10.997	(−6.62, 37)	0.17		
	Statins	20.566	10.735	(−0.72, 41.85)	0.058		

MAFLD: 0-no, 1-yes; Creatinine: 0-normal; 1-high (>1.3 mg/dL for males, >1.1 mg/dL for females); GGT: 0-normal; 1-high (>55 UI/L for males, >33 UI/L for females); Statins: 0-no, 1-yes. BMI, body mass index; HbA1c, glycosylated hemoglobin; MAFLD, metabolic-associated fatty liver disease; GGT, gamma-glutamyl transferase.

## Data Availability

All data presented in this study are reported in this manuscript or available in the Supplementary Materials.

## Supplementary Materials

The following supporting information can be downloaded at: https://www.mdpi.com/article/10.3390/jcm12031013/s1, Table S1: Clinical characteristics of the normotriglyceridemic, non-obese patients; Table S2: List of primers used in the study.
