# Supplementary material for: FABP4 Expression in Subcutaneous Adipose Tissue Is Independently Associated with Circulating Triglycerides in Obesity
Source: J Clin Med. 2023 Jan 28;12(3):1013. doi: 10.3390/jcm12031013 (PMC9917808; doi:10.3390/jcm12031013)
Supplement: Supplementary file 1 [file jcm-12-01013-s001.zip › jcm-2142736-supplementary.pdf]

Supplementary Table S1. Clinical characteristics of the normotriglyceridemic, non-obese patients.

	**Non-Ob (n = 21)**
Women	13 (62%)
Age (years)	60.81 ± 9.34
Weight (kg)	74 (66.50-82.00)
Height (m)	1.67 (1.54-1.73)
BMI (kg/m^2)	27.76 (25.91-28.87)
CUN-BAE Index	36.16 (30.79-42.09)
FPG	93 (84-98)
HbA1c (%)	5.30 (4.60-7.60)
T2D	2 (10%)
T2D medications	2 (10%)
Insulin treatment	1 (5%)
HTN	4 (19%)
HTN medications	4 (19%)
AST (UI/l)	20 (18-27)
ALT(UI/l)	18 (15-36)
GGT (UI/l)	27 (20-44)
AST:ALT ratio	1.00 (0.58-1.42)
MAFLD	2 (10%)
FIB4 score	1.15 (0.84-1.29)
APRI score	0.20 (0.16-0.33)
HSI Index	37.72 (34.12-40.53)
TyG Index	4.53 ± 0.27
Total colesterol (mg/dl)	27 (20-44)
TG (mg/dl)	94 (73-136)
HDL (mg/dl)	60.05 (55.00-84.00)
LDL (mg/dl)	104.5 ± 33.53
Statins treatment	2 (10%)
Creatinine (mg/dl)	0.77 (0.63-0.87)

Data are presented as the mean ± SD, median (IQR) or number (%). CUN-BAE Index, body adiposity estimator; hs-CRP, high-sensitivity C-reactive protein; FPG, fasting plasma glucose; HbA1c, glycosylated haemoglobin; T2D, type 2 diabetes; HTN, hypertension; AST, serum aspartate aminotransferase level; ALT, serum alanine aminotransferase; GGT, gamma-glutamyl transferase; MAFLD, metabolic-associated fatty liver disease; FIB-4, index for liver fibrosis; APRI, AST to platelet ratio index; HSI, hepatic steatosis index; TyG, triglyceride glucose index; FLI, fatty liver index; TG, serum triglycerides; HDL, serum high-density lipoprotein cholesterol; LDL, serum low-density lipoprotein cholesterol.

Supplementary Table S2. List of primers used in the study.

GENE	SEQUENCE	(5'-3')
ABCA1	F	GGAGGCCAGAATGACATCTTAG
ABCA1	R	TTTCCAGCCCCATTAACTCC
ACOX1	F	ACCATTGCCATCCGATACAG
ACOX1	R	GGTCTCCTTCATGTATGCGC
ADFP/PLIN2	F	AGTATCCCTACCTGAAGTCTGTG
ADFP/PLIN2	R	CCCCTTACAGGCATAGGTATTG
ADIPOQ	F	ACAATGACTCCACCTTCACAG
ADIPOQ	R	TTCCTAACCGTACTGAAAGCC
ADIPOR1	F	TCATCTACCTCTCCATCGTCTG
ADIPOR1	R	CACTCAAGCCAAGTCCCAG
ADIPOR2	F	CAGCCATTATAGTCTCCCAGTG
ADIPOR2	R	CCGAGATGACATAGTGCAAGG
ADRB1	F	CCGGGAACAGGAACACAC
ADRB1	R	GAAAGCAAAAGGAAATATGTCTTGA
ADRB3	F	TTTTCTAAACCCCAGCCTTG
ADRB3	R	CACGGCACCTGGACACTAC
ANGPT1	F	TTAAAGGACTTACAGGGACAGC
ANGPT1	R	GACCACATGCATCAAACCAC
ANGPT2	F	CCACGAGACTTGAACTTCAGC
ANGPT2	R	TGTGCTTGTCTTCCATAGCTAG
ATG12	F	AATCAGTCCTTTGCTCCTTCC
ATG12	R	GCAAGTTGATTTTCTTTGTGGTTC
ATG5	F	AGCAACTCTGGATGGGATTG
ATG5	R	AGGTCTTTCAGTCGTTGTCTG
ATG7	F	TTTTGCTATCCTGCCCTCTG
ATG7	R	GCTGTGACTCCTTCTGTTTGAC
ATGL	F	CACTTCAACTCCAAGGACGAG
ATGL	R	CTCATAGAGTGGCAGGTTGTC
CD14	F	CAGAGGTTCGGAAGACTTATCG
CD14	R	TTCGGAGAAGTTGCAGACG
CD206/MRC1	F	GCAAAGTGGATTACGTGTCTTG
CD206/MRC1	R	CTGTTATGTCGCTGGCAAATG
CD68	F	ATGGCGGTGGAGTACAATG
CD68	R	TGGACAGCTGGTGAAAGAATG
CD80	F	CCATCCAAGTGTCCATACCTC
CD80	F	GCCAGCTCTTCAACAGAAAC
CPT1A	F	TCCAGTTGGCTTATCGTGGTG
CPT1A	R	CTAACGAGGGGTCGATCTTGG
DGAT2	F	TCCGAATGCCTGTGTTGAG
DGAT2	R	CAAATAGTCTATGGTGTCCCGG
DIO2	F	TCCAGTGTGGTGCATGTCTC
DIO2	R	CTGGCTCGTGAAAGGAGGTC
F13A1	F	ATCCCATCAAAGTCAGCCG
F13A1	R	ATGTCAACGCTTCCAGTCC
FABP4	F	CATGTGCAGAAATGGGATGG
FABP4	R	AACTTCAGTCCAGGTCAACG
FASN	F	CAGAGTCGGAGAACTTGCAG
FASN	R	GGAGGCATCAAACCTAGACAG
HIF1A	F	AAGAACTTTTAGGCCGCTCA
HIF1A	R	CAACCCAGACATATCCACCTC
HSL/LIPE	F	TCATCTCCATCGACTACTCCC
HSL/LIPE	R	AGATTCGTTCCCCTGTTGAG
IL6	F	CAACCTGAACCTTCCAAAGATG
IL6	R	ACCTCAAACTCCAAAAGACCAG
IRS1	F	TCTGCTCAGCGTTGGTG
IRS1	R	GTGCATGCTCTTGGGTTTG
LEP	F	GCTTCAGGCTACTCCACAG
LEP	R	CCTTCCCTTAACGTAGTCCTTG
LEPR	F	TCAACCAGTACAATCCAGTCAC
LEPR	R	TTTGGGCTCAGATATGGGATG
LPL	F	GGACTGAGAGTGAAACCCATAC
LPL	R	GGAAGGAGTAGGTCTTATTTGTGG
MCP1/CCL2	F	CCTCCAGCATGAAAGTCTCTG
MCP1/CCL2	R	TCTGCACTGAGATCTTCCTATTG
MGLL	F	AGCATGCCAGAGGAAAGTTC
MGLL	R	ATGGGACACAAAGATGAGGG
MMP13	F	GGAATTGGTGATAAAGTAGATGCTG
MMP13	R	ACGCGAACAATACGGTTACTC
MMP14	F	GGATGGACACGGAGAATTTTG
MMP14	R	TTTATCAGGAACAGAAGGCCG
MMP15	F	CCCAAGCCCATCAGTGTC
MMP15	R	CGCTCATTGTCGAATTTCCAG
MOGAT1	F	GAAAGCCATCCACACTGTTG
MOGAT1	R	GCCATACTTTCCTTTGTGTTCC
MSR1	F	ATCTGTGAAATTTGATGCTCGC
MSR1	R	CCAATGAGAGGGATGAGAACTG
P16/CDKN2A	F	GATGTCGCACGGTACCTG
P16/CDKN2A	R	TCTCTGGTTCTTTCAATCGGG
P21/CDKN1A	F	GAACTTCGACTTTGTCACCGAGAC
P21/CDKN1A	R	TGGAGTGGTAGAAATCTGTCATGCT
P53/TP53	F	CAGCACATGACGGAGGTTGT
P53/TP53	R	TCATCCAAATACTCCACACGC
PAI-1/SERPINE1	F	GTGGACTTTTCAGAGGTGGAG
PAI-1/SERPINE1	R	GAAGTAGAGGGCATTCACCAG
PDGFRA	F	TTCCTCTGCCTGACATTGAC
PDGFRA	R	GTCTTCAATGGTCTCGTCCTC
PDGFRB	F	ATGTGACGGAGAGTGTGAATG
PDGFRB	R	GCAGCTCAGCAAATTGTAGTG
PGC1B	F	GTACATTCAAAATCTCTCCAGCGACAT
PGC1B	R	GAGGGCTCGTTGCGCTTCCTCAGGGC
PLIN1	F	CATTGAGAAGGTGGTGGAGTAC
PLIN1	R	GTGTATCGAGAGAGGGTGTTG
PLIN2	F	AGTATCCCTACCTGAAGTCTGTG
PLIN2	R	CCCCTTACAGGCATAGGTATTG
PPARA	F	CTATCATTTGCTGTGGAGATCG
PPARA	R	AAGATATCGTCCGGGTGGTT
PPARG	F	GTCGGTTTCAGAAATGCCTTG
PPARG	R	GCTGGTCGATATCACTGGAG
PPARGC1A/PGC1A	F	CAGGCAGTAGATCCTCTTCAAG
PPARGC1A/PGC1A	R	TCCTCGTAGCTGTCATACCTG
PTEN	F	TTTGAAGACCATAACCCACCAC
PTEN	R	ATTACACCAGTTCGTCCCTTTC
RPL6	F	CCTTAATTCTCTTTCCCATCTTGC
RPL6	R	TTCTTGGCTTCGGGTTTCTT
SLC2A1/GLUT1	F	TCATCGTGGCTGAACTCTTC
SLC2A1/GLUT1	R	GATGAAGACGTAGGGACCAC
SLC2A4/GLUT4	F	ACTGGACGAGCAACTTCATC
SLC2A4/GLUT4	R	GAGGACCGCAAATAGAAGGAA
SOD2	F	GACAAACCTCAGCCCTAACG
SOD2	R	GAAACCAAGCCAACCCCAAC
SREBF1	F	TTCTGACAGCCATGAAGACAG
SREBF1	R	CCGCATCTACGACCAGTG
TGFB1	F	TTGATGTCACCGGAGTTGTG
TGFB1	R	GTAGTGAACCCGTTGATGTCC
TNFA	F	AGGTCTACTTTGGGATCATTGC
TNFA	R	GAAGAGGTTGAGGGTGTCTG
UCP1	F	GGACTACTCCCAATCTGATGAG
UCP1	R	AAATCCAGCGATAAGAGCCG
UCP2	F	TCCTGAAAGCCAACCTCATG
UCP2	R	GGCAGAGTTCATGTATCTCGTC
UCP3	F	AGAAAATACAGCGGGACTATGG
UCP3	R	CTTGAGGATGTCGTAGGTCAC
VEGFA	F	AGTCCAACATCACCATGCAG
VEGFA	R	TTCCCTTTCCTCGAACTGATTT
VEGFB	F	CTTAGAGCTCAACCCAGACAC
VEGFB	R	ACCCTGCTGAGTCTGAAAAG
VEGFR1	F	TCCCTCAACCTACAATCAAGTG
VEGFR1	R	GCTCTCAATTCTGTTTCCCATG
VEGFR2	F	CATTTCAAAGGAGAAGCAGAGC
VEGFR2	R	GAGGAATGGCATAGACCGTAC

F, forward; R, reverse.
